# Network analysis of DSM-5 criteria for gambling disorder: considering sex differences in a large clinical sample

**DOI:** 10.1192/j.eurpsy.2024.22

**Published:** 2024-05-06

**Authors:** Ignacio Lucas, Bernat Mora-Maltas, Roser Granero, Zsolt Demetrovics, Víctor Ciudad-Fernández, Giovanna Nigro, Marina Cosenza, Magda Rosinska, Javier Tapia, Fernando Fernández-Aranda, Susana Jiménez-Murcia

**Affiliations:** 1 Clinical Psychology Department, Bellvitge University Hospital, Barcelona, Spain; 2 Psychoneurobiology of Eating and Addictive Behaviours Group, Bellvitge Biomedical Research Institute (IDIBELL), Barcelona, Spain; 3 CIBER de Fisiopatología de la Obesidad y Nutrición (CIBERobn), Instituto de Salud Carlos III, Barcelona, Spain; 4 Departament de Psicobiologia i Metodologia, Universitat Autònoma de Barcelona, Bellaterra, Spain; 5 Institute of Psychology, ELTE Eötvös Loránd University, Budapest, Hungary; 6 Centre of Excellence in Responsible Gaming, University of Gibraltar, Gibraltar, Gibraltar; 7 Department of Personality, Assessment and Psychological Treatment, Faculty of Psychology, University of València, Valencia, Spain; 8 Department of Psychology, Università degli studi della Campania “Luigi Vanvitelli”, Viale Ellittico, Caserta, Italy; 9 Body Image Assessment and Intervention Unit, Department of Clinical Health and Psychology, Autonomous University of Barcelona, Barcelona, Spain; 10 Gerencia Territorial Metropolitana Sud. Hospital Universitari de Bellvitge, Barcelona, Spain; 11 Department of Clinical Sciences, School of Medicine and Health Sciences, University of Barcelona, Barcelona, Spain

**Keywords:** chasing losses, gambling disorder, network analysis, tolerance, withdrawal

## Abstract

**Background:**

The fifth version of the Diagnostic and Statistical Manual for Mental Disorders (DSM-5) and its revised version (DSM-5-TR) propose severity levels for gambling disorder (GD) based on the number of criteria met. However, this taxonomy has some limitations. We aimed to assess the centrality of each criterion and its relationship by conducting a network analysis while considering sex differences.

**Methods:**

We performed a network analysis with the DSM-5 criteria for GD with data from 4,203 treatment-seeking patients (3,836 men and 367 women) diagnosed with GD who sought for treatment in a general tertiary hospital which has a unit specialized in behavioral addictions.

**Results:**

The withdrawal criterion (“Restless or irritable when attempting to cut down or stop gambling”) showed the highest centrality values in both sexes. In men, the second most central criterion was the tolerance criterion (“Needs to gamble with increasing amounts of money in order to achieve the desired excitement”); while among women, the second was the chasing losses criterion (“After losing money gambling, often returns another day to get even”).

**Conclusions:**

The most central criteria identified are associated with compulsivity-driven behaviors of the addictive process. Taking into account the high relevance and transitive capacity of withdrawal in both men and women, as well as tolerance in men, and chasing losses in women, the recognition and understanding of these symptoms are fundamental for the accurate diagnosis and severity assessment of GD.

## Introduction

Gambling disorder (GD) is the only behavioral addiction (BA) included in the main section of the fifth version of the Diagnostic and Statistical Manual for Mental Disorders (DSM-5) [1] and the DSM-5-TR [2]. The fifth edition of the DSM introduced changes aimed to improve the diagnostic accuracy of GD [3–5]. For instance, GD was relocated from the Impulse-Control Disorders Not Classified Elsewhere category to the Substance-Related and Addictive Disorders category, the illegal acts criterion was removed [6, 7], and the threshold of diagnosis was reduced from five to four criteria [8–10]. However, while the International Classification of Diseases [11] distinguishes between essential and additional features of GD, the DSM-5 gives the same significance to all the symptoms, taking into account only the number of fulfilled criteria. Furthermore, in contrast to categorical approaches, other models such as The Research Domain Criteria (RDoC) from the National Institute of Mental Health [12] and the Hierarchical Taxonomy of Psychopathology (HiTOP) [13] propose dimensional frameworks for the study of psychopathology, including GD. The DSM-5 integrated this dimensional approach, resulting in the inclusion of severity ratings for GD. Replicating the severity classification for Substance Use Disorders (SUD), three severity categories were proposed for GD, depending on the number of criteria met: mild (4 or 5 criteria), moderate (6 or 7) or severe (8 or 9) [1, 2]. For SUD, the number of fulfilled criteria has proven to be a good severity indicator [14]. However, for GD, this taxonomy showed some limitations regarding the lack of significant differences between the moderate and severe categories in terms of psychopathology and functional impact. Also, no differences have been observed in terms of treatment outcome between the three categories of severity [15, 16]. One possible reason for these limitations could be that each diagnostic criterion may have a different level of significance to the disorder [8, 17, 18]. In the same vein, an increase in the weight of the most central criteria could improve the accuracy of the severity level diagnosis for GD [8, 17]. Thus, it would be important to determine the core criteria that have a stronger influence on GD severity, as proposed for other BAs [19–21]. However, there is no clear agreement among researchers as to which would be the core criteria of GD. One recognized model of addiction, the “components” model, proposes six core features: salience, mood modification, tolerance, withdrawal, conflict, and relapse [22, 23]. However, others argue that the addiction process, rather than the symptoms, should be the primary focus because it serves as the foundation for symptom development and maintenance [20, 24]. In this regard, the transition from impulsive-related behaviors (positive reinforcement) to later compulsivity-driven behaviors (negative reinforcement) have been described as one of the key mechanisms underlying addiction [25, 26]. This crossover from goal-directed to compulsive behavior has also been described for BA [27–29]. These later compulsive motives may eventually produce withdrawal syndrome/negative affect when the objective cannot be achieved [30]. Therefore, the GD criteria associated with the promotion of the addictive process through negative reinforcement, such as withdrawal, may be directly related to the course and severity of the addictive process [31]. Moreover, several studies have suggested that withdrawal would be one of the symptoms most closely related to the severity of GD [17, 18, 32–34].

In addition, when describing the more central features of GD severity, it is essential to consider the differences between men and women [35–37]. Although studies about GD in women are scarce [38], the literature reports that women present more of a preference than men for nonstrategic gambling forms (e.g., lottery or slot machines), have a lower socioeconomic status, and have higher psychopathology related to comorbidities, such as affective disorders [5, 35, 36, 39–42]. In general, women tend to use gambling more as a way to regulate their emotional state [7, 43–46], and men tend to use it more as a thrilling activity [47]. In summary, these distinctions may constitute differences in terms of the weight of each criterion between women and men.

With the objective of defining the weight and relationship of each criterion, network analysis (NA) is an appropriate approach to determine the spatial/functional structures of psychological constructs based on the relevance and relationships of their features [48, 49]. In clinical research, NA has already been used to determine the relevance of each symptom and their interconnection for different psychopathological conditions such as depression [50], post-traumatic stress disorder [51], eating disorders [52, 53], and addictive disorders [49, 54–64].

In addictive disorders, NA has already yielded interesting results about the relevance and relationship of the symptoms. Analysis of the centrality and connections of SUD symptoms across different substance classes determined that the highest centrality for using a substance more than planned had a strong interaction with tolerance [49]. In the specific case of alcohol use disorder, loss of control [57] and physiological dependence (withdrawal) have been reported as the most central features [54]. Likewise, other research studies have analyzed the factors of multiple substances and BAs using NA, finding unique features for each taxonomy [58].

Some studies also showed the utility of NA in determining the centrality of the symptoms in different types of BA. For instance, the most central features of internet gaming disorder were conflict, withdrawal, and tolerance [59], whereas for problematic smartphone use, these were loss of control and continued excessive use [60]. Regarding problematic pornography use, these were salience, mood modification, and withdrawal [56], and for problematic social media, they were problems in self-regulation and preference for online communication [61]. However, in line with the differences found between SUD and different types of BA [58], NA of potentially addictive behaviors also suggests that different internet-based behaviors should be considered as separate entities, with specific features for each activity [55, 62, 64]. This evidence emphasizes the necessity of analyzing the centrality of the specific symptoms related to each type of BA. On the basis of these results, GD should be analyzed independently from other types of BA. Furthermore, NA of problematic gambling in women showed more association with gambling machines, while in men it was more associated with sports betting, poker, and casino games [63], consistent with the higher preference for strategic gambling in men and nonstrategic gambling in women [65]. In this regard, to our knowledge, no study has used an NA approach to examine the relevance and interconnections of each GD criteria of the DSM-5 in a large sample of treatment-seeking patients with GD, considering differences between men and women.

### Aims and hypotheses

The aim of this study was to use the NA approach to determine the centrality of each DSM-5 criterion for GD in a large clinical sample, with a special focus on sex differences. In this regard, criteria that are directly related to the negative reinforcement process, such as withdrawal, could have more relevance and influence in the co-occurrence of other symptoms. Moreover, bearing in mind the differences that have been described between men and women diagnosed with GD, we hypothesize that both sexes would present different key symptoms.

## Method

### Participants

The sample was composed of 4,203 patients (3,836 men and 367 women) diagnosed with GD. All of them sought treatment at the BAs Unit of the University Hospital of Bellvitge, a public hospital in Spain certified as a tertiary care center for the treatment of GD. The recruitment process took place between January 2005 and March 2023. They were evaluated by experienced clinical psychologists in two sessions before the start of treatment. During the first session, the clinical psychologist conducted a semi-structured interview to confirm the diagnosis of GD and explored various aspects of gambling behavior and sociodemographic data, including age, age at onset of the GD, duration of GD, marital status, highest academic level achieved, employment situation, personal income, and family income (social position was calculated by the Hollingshead’s index [66]). During this first session, they also signed the informed consent form to participate in the study. During the second assessment session, participants completed a battery of validated psychometric instruments, including the Diagnostic Questionnaire for Pathological Gambling according to the DSM criteria [9, 10]. All patients had a diagnosis of GD according to DSM-5 criteria (≥4 criteria). This study was carried out in accordance with the Declaration of Helsinki. The University Hospital of Bellvitge’s Ethics Committee of Clinical Research approved the study (Refs. 34/05, 307/06).

### DSM-5 criteria

Diagnostic criteria for GD (Table 1) were assessed before the start of treatment using the Spanish adaptation of the Diagnostic Questionnaire for Pathological Gambling [9, 10]. This instrument has shown satisfactory reliability and validity. It should be noted that with the release of the DSM-5, pathological gambling was reclassified and renamed as GD. Therefore, all patients’ diagnoses were re-evaluated and recodified *post hoc* according to the DSM-5 criteria. This instrument is a self-report measure composed of 19 items coded on a binary scale (Yes/No). The internal consistency for this study was α = .761.

**Table 1.** DSM-5 and DSM-5-TR diagnostic criteria for gambling disorder

A1. Needs to gamble with increasing amounts of money in order to achieve the desired excitement.
A2. Is restless or irritable when attempting to cut down or stop gambling.
A3. Has made repeated unsuccessful efforts to control, cut back, or stop gambling.
A4. Is often preoccupied with gambling (e.g., having persistent thoughts of reliving past gambling experiences, handicapping or planning the next venture, thinking of ways to get money with which to gamble).
A5. Often gambles when feeling distressed (e.g., helpless, guilty, anxious, depressed).
A6. After losing money gambling, often returns another day to get even (“chasing” one’s losses).
A7. Lies to conceal the extent of involvement with gambling.
A8. Has jeopardized or lost a significant relationship, job, or educational or career opportunity because of gambling.
A9. Relies on others to provide money to relieve desperate financial situations caused by gambling.

*Note:* Severity: mild (four or five criteria), moderate (six or seven criteria), and severe (eight or nine criteria). Extracted from DSM-5 (APA, 2013) and DSM-5-TR (APA, 2022).

### Statistical analysis

Stata18 for Windows was used for the analysis of the sociodemographic data [67], with chi-square analysis for categorical variables and t-test for quantitative measures. The Gephi 9.2 for Windows program was used to obtain the network in this work [68] (available at http://gephi.org). This statistical software has been specifically developed for exploring and visualizing networks within diverse datasets, and it allows a powerful spatialization process and the computation of the essential parameters of centrality, linkage, and density. In this work, each node represents a DSM-5 criterion for GD and the edges of the underlying relationship pattern. The centrality indices calculated for the nodes provide a measure of the relevance of each criterion, while the linkage indices can be interpreted as the transitive capacity of each node toward the co-occurrence of the other criteria. The analysis was not preregistered and the results should be considered exploratory.

Two separate networks were visualized in this study, collected from subsamples of men and women. The weights of the edges (the effect size and the signal [indicating positive versus negative relationships]) were calculated as the partial correlation coefficient between each of the two nodes, adjusted for the rest of the nodes. This correlation matrix provided the specific degree of association between the two DSM-5 criteria, controlling the potential effect of the other DSM-5 criteria, which were removed. The initial data structure for the network resulted in 9 nodes and 36 potential edges, some of which had very low weights (partial correlations around 0). To simplify this initial complex structure, as usual in NA, only edges that reached significance (p < .05) were modeled.

The relevance and linkage capacity of the nodes were measured through two centrality indices [69]: a) eigenvector centrality, which provided the relative prominence of each node based on the weighted sum of centrality measures of all nodes connected to a node; and b) closeness centrality, which provided the relative connection capacity based on how close the node is to all the other nodes in the graph (these values are calculated as the reciprocal of the sum of the length of the shortest paths between the node and all other nodes in the graphon). High eigenvector centrality indicated that the information contained in a specific node is highly valuable for the entire graph. High closeness centrality indicated a short average distance between one node and all the other nodes (these nodes have a high capacity to promote relevant changes in other areas of the network structure).

In addition to the centrality measures, other indices interpreted in the study were as follows: a) the (average) path length, calculated as the mean of the shortest paths between all pairs of nodes (this value represents a measure of the efficiency of information transport in the network); and b) the diameter, calculated as the greatest distance between the two furthest nodes (representing the maximum eccentricity of any vertex in the graph) [70]. The density of the graph was also estimated as the number of connections divided by the number of possible connections, which provides a measure of how close the network is to being complete (a complete graph includes all possible edges and achieves a density measure equal to 1).

## Results

### Sociodemographic data

Table 2 presents the distribution and differences in sociodemographic features between the subsamples of women and men. The sample of men was younger than women (41.41 [SD = 12.81] versus 50.18 [SD = 13.45] years old). Same for the age of GD onset (29.22 [SD = 12.29] years for men, 37.48 [SD = 11.63] for women). Both groups showed no differences in the duration of the GD. Mean personal and family incomes were higher in the sample of men (1248.02 and 2122.30 euros, respectively) than in the sample of women (898.39 and 1691.16). There were differences in the distribution of marital status, employment, and social position between men and women. Women had higher rates of divorce, unemployment, and lower social status. No differences were observed in their education level.

**Table 2.** Sociodemographic data of the sample

	Women		Men			
	N = 367		N = 3,836			
	Mean	SD	Mean	SD	p	η^2^
Age (yrs)	50.18	12.81	41.41	13.45	<.001*	.033
Age of onset of GD (yrs)	37.48	12.29	29.22	11.63	<.001*	.038
Duration of GD (yrs)	6.08	6.11	6.15	6.10	.846	.001
Income (euros) personal	898.39	742.10	1248.02	976.35	<.001*	.011
Family	1691.16	1288.49	2122.30	1499.75	<.001*	.007
	n	%	n	%	p	V
Marital status single	157	42.8%	1609	41.9%	<.001*	.067
Married – couple	135	36.8%	1727	45.0%		
Divorced – separated	75	20.4%	500	13.0%		
Education primary	227	61.9%	2209	57.6%	.210	.027
Secondary	115	31.3%	1379	35.9%		
University	25	6.8%	248	6.5%		
Employment unemployed	196	53.4%	1595	41.6%	<.001*	.068
Employed	171	46.6%	2241	58.4%		
Social position index high	3	.8%	61	1.6%	<.001*	.099
Mean–high	11	3.0%	190	5.0%		
Mean	42	11.4%	396	10.3%		
Mean–low	71	19.3%	1280	33.4%		
Low	240	65.4%	1909	49.8%		

*Note.*GD: gambling disorder. SD: standard deviation. V: Cramer’s V coefficient. η^2^: Eta-squared coefficient.

### DSM-5 criteria distribution

Table 3 displays the prevalence of each DSM-5 criterion within the women and men subsamples, as well as the proportion comparisons. The most frequent criterion was A7 (“lies related to gambling activity”) (95.1% of women reported this behavior and 94.3% of men; *p* = .536). The least frequent criterion was A1 “gambling with an increasing amount of money” (63.2% of women reported this behavior and 62.5% of men; *p* = .798). Differences between sexes were found for A3 “lack of control” (more frequent among men), A5 “gamble as a way of escaping” (more frequent among women”) and A8 “social impact” (more frequent among men).

**Table 3.** Distribution of the DSM-5 criteria for GD in the study

	Women		Men			
	N = 367		N = 3,836			
	*n*	%	*n*	%	*p*	*V*
A1. Gambling with increasing amount–money (“tolerance”)	232	63.2%	2,399	62.5%	.798	.004
A2. Withdrawal	273	74.4%	2,938	76.6%	.342	.015
A3. Lack of control	324	88.3%	3,539	92.3%	.008*	.041
A4. Preoccupied	248	67.6%	2,426	63.2%	.099	.025
A5. Gamble as a way of escaping	328	89.4%	2,667	69.5%	.001*	.124
A6. After losing returns (“chasing” one’s losses)	301	82.0%	3,225	84.1%	.306	.016
A7. Lies related to gambling	349	95.1%	3,618	94.3%	.536	.010
A8. Social impact	299	81.5%	3,295	85.9%	.021*	.035
A9. Relies on others to provide money	271	73.8%	2,953	77.0%	.174	.021
	*Mean*	*SD*	*Mean*	*SD*	*p*	η^2^
DSM–5 Total number of criteria	7.18	1.62	7.10	1.60	.381	.001

*Note.* SD: standard deviation. V: Cramer’s V coefficient. η^2^: Eta-squared coefficient. Comparison between the prevalences based on chi-square tests, and comparison between means based on T-test.

Table S1 (supplementary material) contains the prevalence of the DSM-5 criteria stratified (separately) by sex and by the GD severity group.

### Network analysis

**Figure 1.**
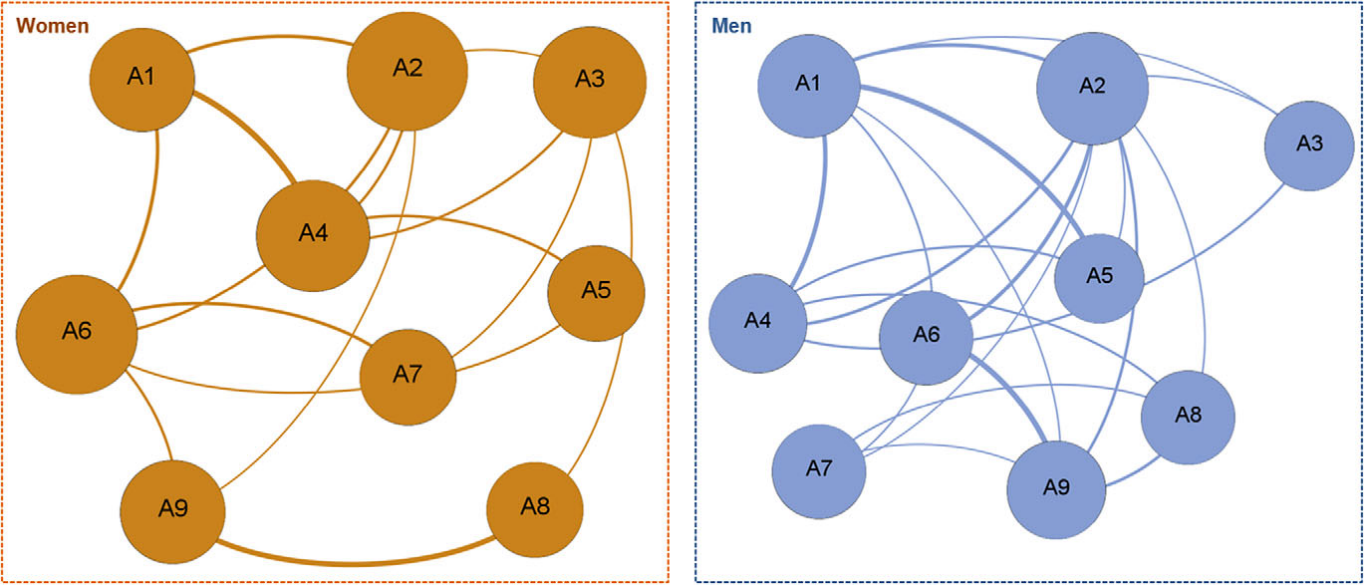
Visualization of the networks among women (left) and men (right) subsamples. *Note.* Edge thickness represents the relative edge weight strength. Node size represents the relative weight in the network. All the edges obtained a positive signal.

**Figure 2.**
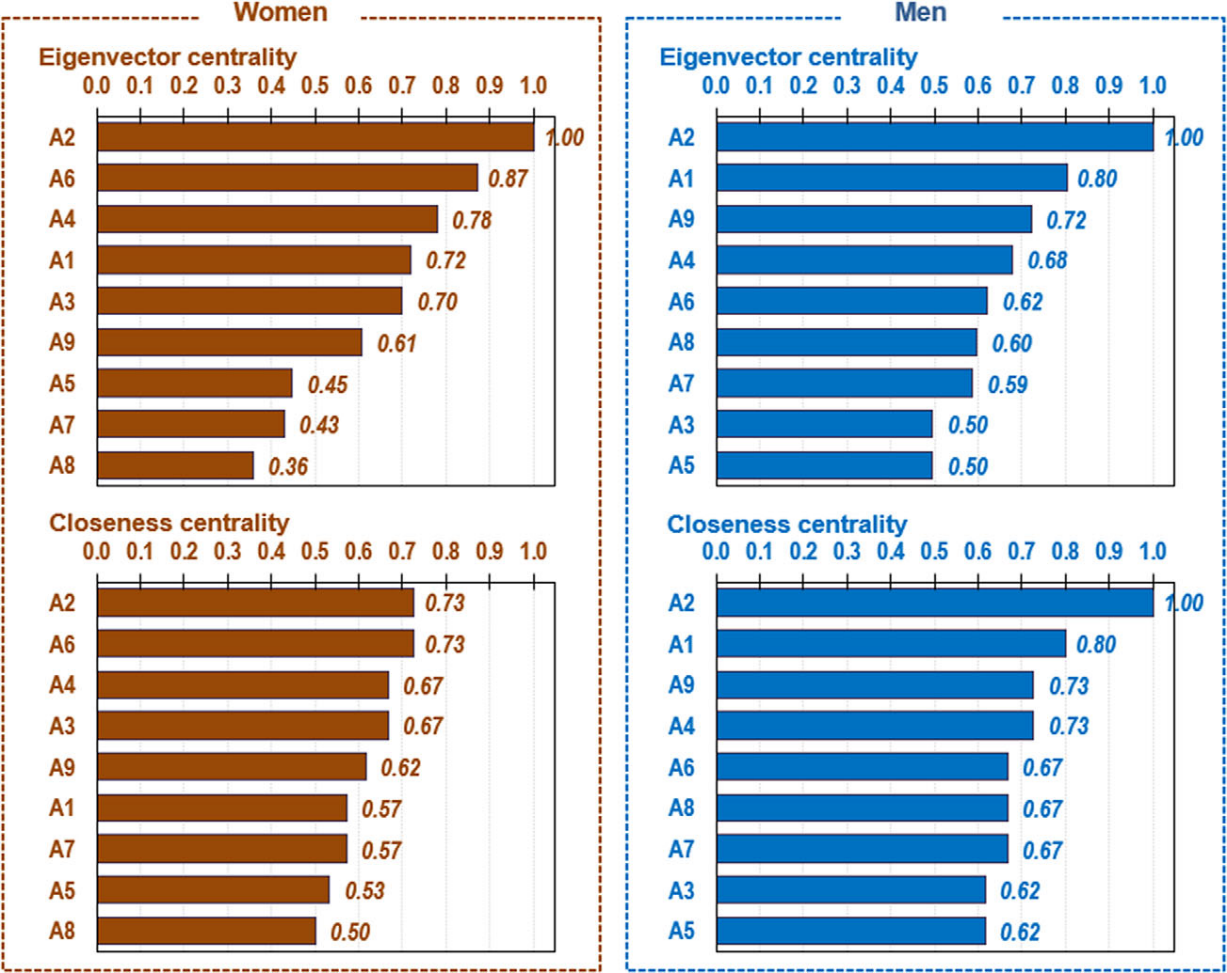
Relevance of centrality and linkage of the nodes among women (left) and men (right) subsamples.

The first panel of Figure 1 displays the visualization of the network obtained among the women subsample, and the left panel of Figure 2 displays the bar charts with the nodes ordered according to the eigenvector and the closeness centrality. The network for women achieved a density equal to 0.417 (around 42% of the potential edges were modeled), an average path length equal to 1.639, and a diameter equal to 3.0. According to the eigenvector centrality indices, the node with the highest relevance in the network was A2 “withdrawal” (this specific DSM-5 criterion was identified as the behavior with the greatest influence in the graphon, with an eigenvector centrality equal to 1). According to the closeness centrality, the highest linkage capacity was achieved by A2 “withdrawal” and A6 “chasing one’s losses” (the activation of these specific DSM-5 criteria, which achieved a closeness coefficient equal to 0.73, had the greatest impact on the other nodes).

The network obtained among the subsample of men (the right panel of Figure 1) achieved a density equal to 0.583 (resulting in 58.3% of the potential edges modeled), an average path length equal to 1.417, and a diameter equal to 2.0. The centrality indices (the right panel of Figure 2) indicated that A2 “withdrawal” was the DSM-5 criterion with the highest relevance and linkage capacity (both eigenvector and closeness centrality indexes achieved a value equal to 1).

Table S2 (supplementary material) contains the complete results obtained in the NA among women and men subsamples.

## Discussion

This study explored the network structure of the GD criteria defined by the DSM-5 taxonomy in a large sample of treatment-seeking patients with GD, considering the differences between men and women. The NA results reported that withdrawal criterion (“Restless or irritable when attempting to cut down or stop gambling”) had the highest centrality values, regardless of sex. This result confirms our initial hypothesis about withdrawal being closely related to the course and severity of the addictive process [31], and fits with previous literature that emphasized the relevance of withdrawal to the severity of GD [17, 18, 32–34]. This might indicate that the gambling addiction process could be driven by compulsive motives with the aim of avoiding the discomfort associated with not gambling (negative reinforcement) [27–29], and suggest that, if the patient reports withdrawal, they may be more likely to also present with other GD criteria and, following the definition of the DSM-5, present greater severity of the disorder.

Regarding our second hypothesis, the rest of the hierarchy extracted from the NA reported differences between the sexes. Women and men differ in the second core node. In the sample of men diagnosed with GD, the tolerance symptom (“Needs to gamble with increasing amounts of money in order to achieve the desired excitement”) is the second most relevant and transitive criterion of the network. In contrast, in the sample of women diagnosed with GD, the chasing losses criterion (“After losing money gambling, often returns another day to get even” (“chasing” one’s losses) is the second most central criterion. These findings fit with previous longitudinal data having related tolerance and chasing losses with a more severe progression of GD [71]. It might be possible that the relevance of chasing losses in women would be affected by their socioeconomic status [72]. In our sample, women had a lower social position with higher unemployment rates and lower economic income. These factors may produce a stigma that emphasizes the relevance of trying to recover money through gambling due to the higher impact of incurring economic losses [72]. In addition, it should be noted that tolerance and chasing one’s losses have been seen to be closely related, as the latter could be a different form of expression of tolerance [30], perhaps a more planned one.

Previous literature has already reported that the DSM-5 severity classification for GD presents important limitations regarding psychopathology, functional impact, and treatment outcome [15, 16]. Moreover, these results show that most patients who seek treatment for GD usually present with moderate or severe forms of the disorder. According to the DSM-5, each criterion would exert the same influence on the severity of the disorder, as in SUD [14]. However, the results presented in this study are in line with previous research that supports the different significance of each GD criterion [8, 17, 18]. In light of these results, more weight should be given to those symptoms that concur with the physiological hallmarks of SUD, withdrawal, and tolerance [32]. Both symptoms would be directly involved in the development of the addictive process and, therefore, in the course and severity of GD [20].

This study provides empirical evidence of the importance of withdrawal and tolerance in GD severity [34]. The conceptualization of withdrawal and tolerance as core features of GD severity would comply with the addiction models that highlight the importance of the “components” [22, 23], as these criteria are considered core features of the addiction. In addition, there are proposals that focus on the process of addiction [20, 24], as these criteria may be directly related to the transition from goal-directed behaviors to compulsivity-driven behaviors [27–29]. However, although negative reinforcement processes have been historically associated with the development and maintenance of an addiction disorder [73], both withdrawal and tolerance have been criticized in GD and other BA due to the lack of empirical support [24, 74–76]. These findings also reaffirm the need for further research that acknowledges the precise description of withdrawal and tolerance symptoms in GD and their differences with those observed in SUD. For instance, withdrawal symptoms in GD do not have to be analogous to those present in SUD. Most studies that acknowledge the importance of withdrawal in GD have obtained this symptomatology by self-report from the participants [17, 18, 32, 33]. Moreover, regarding tolerance, the necessity to gamble with increased amounts of money to achieve the same excitement could be associated with accumulated debts or erroneous perceptions about gambling [30]. Therefore, more research on withdrawal and tolerance in GD would help to precisely define these processes in GD and clarify their strong influence toward the severity of the disorder. Additionally, these results give rise to consider the relevance of other features that are not yet GD criteria, such as craving, which is associated with GD severity [77].

These results emphasize an important aspect of GD, suggesting that patients who report restlessness or irritability when attempting to reduce or stop gambling may signify more severe cases of GD. Withdrawal symptoms may indicate the need for personalized treatments tailored to address severe GD in clinical practice. Recognizing these symptoms as markers of severity underscores the importance of distinguishing between varying degrees of GD and implementing targeted interventions for more effective support. In this line, the dimensional approach already proposed by models such as RDoC [12] and HiTOP [13] could be a promising avenue for studying the clinical features of GD [78]. Just as DSM-5 revised its diagnostic criteria for GD to improve diagnostic accuracy, future editions of the diagnostic manual should consider the relevance of each criterion to determine the severity of GD.

This study is not exempt from limitations. First, the cross-sectional design does not allow for the temporal sequence to be demonstrated in the hierarchy which was extracted from these results. Longitudinal data would be necessary to test whether the presence of one criterion would predict the future development of additional symptomatology. Second, although sex differences were considered, not all existing gambling profiles were assessed, to which the significance of the criteria may vary (e.g., gambling preference, age, impulsivity traits). Third, the absence of control over possible complementary pharmacological treatment. Finally, the sample was non-probabilistic and intentional because data were collected from patients with GD who sought treatment. This makes it difficult to draw conclusions about the entire population with GD.

The study also has several strengths. First, the use of network methodology to describe the structure of the interrelations between the DSM-5 criteria for GD. This analytical approach has rapidly grown in the field of psychopathology during the last decades with promising results. It greatly expands the capacity to easily visualize the dynamics of mental symptoms through a topological explanatory strategy. Network theory underlies the conceptualization of complex psychiatric conditions as the phenomenological manifestation of relatively stable network structures of interacting symptoms. Graph theory provides tools to mathematically quantify the dynamics of the complex systems by their topological properties (i.e., centrality, path length, density). Furthermore, the external validity of these results and their generalization to clinical practice are supported by the use of a large clinical sample of patients formally diagnosed with GD and by the networks obtained for both men and women.

## Conclusions

Defining the relevance and transitional capacity of each criterion may have important implications for the specification of GD severity. Also, defining specific profiles for men and women may help in adapting the criteria to obtain a more precise diagnosis of the disorder. Overall, these results show that certain criteria bear more significance in the severity of GD and, thus, provide additional evidence concerning the limitations of the severity classification for GD proposed in the DSM-5 and the DSM-5-TR. Considering the higher weight of withdrawal in both men and women, as well as tolerance in men and chasing losses in women, such criteria may be helpful in being able to identify the most severe cases of GD. In conclusion, the recognition and understanding of these symptoms are fundamental for the accurate diagnosis of GD, emphasizing their pivotal role in guiding effective treatment strategies and improving patient outcomes.

## Supporting information

Lucas et al. supplementary material Lucas et al. supplementary material

## Data Availability

The datasets generated during and/or analyzed during the current study are not publicly available due to ethical restrictions in order to protect the confidentiality of the participants, but are available from the corresponding author on reasonable request.

## Supplementary material

The supplementary material for this article can be found at http://doi.org/10.1192/j.eurpsy.2024.22.
