# Supplementary material for: Network analysis of DSM-5 criteria for gambling disorder: considering sex differences in a large clinical sample
Source: Eur Psychiatry. 2024 May 6;67(1):e65. doi: 10.1192/j.eurpsy.2024.22 (PMC11536196; doi:10.1192/j.eurpsy.2024.22)
Supplement: Lucas et al. supplementary material [file S0924933824000221sup001.docx]

**SUPPLEMENTARY MATERIAL**

***Table S1.*** *Distribution of the DSM-5 criteria for the different severity categories of GD, for women and men.*

|  | Mild | | Moderate | | Severe | |
| --- | --- | --- | --- | --- | --- | --- |
|  | *N=74* | | *N=107* | | *N=186* | |
| *Women sub-sample* | *n* | *%* | *n* | *%* | *n* | *%* |
| A1. Gambling with increasing amount-money (“tolerance”) | 18 | 24.3% | 52 | 48.6% | 162 | 87.1% |
| A2. Withdrawal | 22 | 29.7% | 73 | 68.2% | 178 | 95.7% |
| A3. Lack of control | 51 | 68.9% | 92 | 86.0% | 181 | 97.3% |
| A4. Preoccupied | 17 | 23.0% | 63 | 58.9% | 168 | 90.3% |
| A5. Gamble as a way of escaping | 53 | 71.6% | 95 | 88.8% | 180 | 96.8% |
| A6. After losing returns (“chasing” one’s losses) | 35 | 47.3% | 86 | 80.4% | 180 | 96.8% |
| A7. Lies related to gambling | 64 | 86.5% | 101 | 94.4% | 184 | 98.9% |
| A8. Social impact | 43 | 58.1% | 76 | 71.0% | 180 | 96.8% |
| A9. Relies on others to provide money | 28 | 37.8% | 72 | 67.3% | 171 | 91.9% |
|  |  | |  | |  | |
|  | Mild | | Moderate | | Severe | |
|  | *N=727* | | *N=1,289* | | *N=1,820* | |
| *Men sub-sample* | *n* | *%* | *n* | *%* | *n* | *%* |
| A1. Gambling with increasing amount-money (“tolerance”) | 124 | 17.1% | 654 | 50.7% | 1621 | 89.1% |
| A2. Withdrawal | 232 | 31.9% | 947 | 73.5% | 1759 | 96.6% |
| A3. Lack of control | 559 | 76.9% | 1196 | 92.8% | 1784 | 98.0% |
| A4. Preoccupied | 171 | 23.5% | 647 | 50.2% | 1608 | 88.4% |
| A5. Gamble as a way of escaping | 262 | 36.0% | 763 | 59.2% | 1642 | 90.2% |
| A6. After losing returns (“chasing” one’s losses) | 394 | 54.2% | 1053 | 81.7% | 1778 | 97.7% |
| A7. Lies related to gambling | 626 | 86.1% | 1205 | 93.5% | 1787 | 98.2% |
| A8. Social impact | 464 | 63.8% | 1061 | 82.3% | 1770 | 97.3% |
| A9. Relies on others to provide money | 310 | 42.6% | 933 | 72.4% | 1710 | 94.0% |

***Table S2 (supplementary).*** *Complete results obtained in the network analysis*

| **Women** | Authority | Eigenvector  centrality | Closeness.  centrality | Harmonic  clos.central. | Betweeness  centrality | HUB | Number of  triangles |
| --- | --- | --- | --- | --- | --- | --- | --- |
| A1 | 0.349919 | 0.719002 | 0.571429 | 0.666667 | 0.333333 | 0.349935 | 2 |
| A2 | 0.485038 | 1.000000 | 0.727273 | 0.812500 | 3.833333 | 0.485046 | 4 |
| A3 | 0.337995 | 0.699932 | 0.666667 | 0.750000 | 5.000000 | 0.337997 | 1 |
| A4 | 0.378443 | 0.778965 | 0.666667 | 0.750000 | 3.250000 | 0.378424 | 2 |
| A5 | 0.217951 | 0.448202 | 0.533333 | 0.604167 | 0.333333 | 0.217971 | 0 |
| A6 | 0.422713 | 0.871342 | 0.727273 | 0.812500 | 6.750000 | 0.422686 | 2 |
| A7 | 0.206955 | 0.428799 | 0.571429 | 0.625000 | 0.500000 | 0.206966 | 0 |
| A8 | 0.171877 | 0.357868 | 0.500000 | 0.583333 | 0.500000 | 0.171872 | 0 |
| A9 | 0.293725 | 0.607555 | 0.615385 | 0.687500 | 2.500000 | 0.293735 | 1 |
|  |  |  |  |  |  |  |  |
| **Men** | Authority | Eigenvector  centrality | Closeness.  centrality | Harmonic  clos.central. | Betweeness  centrality | HUB | Number of  triangles |
| A1 | 0.391277 | 0.802478 | 0.800000 | 0.875000 | 3.166667 | 0.391275 | 8 |
| A2 | 0.487578 | 1.000000 | 1.000000 | 1.000000 | 7.666667 | 0.487575 | 13 |
| A3 | 0.241803 | 0.496014 | 0.615385 | 0.687500 | 0.000000 | 0.241806 | 3 |
| A4 | 0.330622 | 0.678404 | 0.727273 | 0.812500 | 1.666667 | 0.330621 | 6 |
| A5 | 0.241803 | 0.496014 | 0.615385 | 0.687500 | 0.000000 | 0.241806 | 3 |
| A6 | 0.303383 | 0.621528 | 0.666667 | 0.750000 | 0.333333 | 0.303385 | 5 |
| A7 | 0.286720 | 0.587283 | 0.666667 | 0.750000 | 0.333333 | 0.286719 | 5 |
| A8 | 0.291257 | 0.597064 | 0.666667 | 0.750000 | 0.833333 | 0.291258 | 4 |
| A9 | 0.351912 | 0.721138 | 0.727273 | 0.812500 | 1.000000 | 0.351913 | 7 |
